# Development of Robust Ratio Linear Fitting Method of Temperature and Emissivity Separation for High-Temperature Data

**DOI:** 10.3390/s26165151

**Published:** 2026-08-14

**Authors:** Mitchell Manzardo, Michael Dexter, Shannon Young, John Bowlan, Anthony Franz

**Affiliations:** 1 Department of Engineering Physics, Air Force Institute of Technology, 2950 Hobson Way, Wright-Patterson AFB, Dayton, OH 45433, USA; michael.dexter.2@au.af.edu (M.D.); shannon.young.8@au.af.edu (S.Y.); anthony.franz.3@au.af.edu (A.F.); 2 Los Alamos National Laboratory, Los Alamos, NM 87545, USA; jbowlan@lanl.gov

**Keywords:** temperature and emissivity separation, hyperspectral infrared spectroscopy, spectral emissivity, Gray Body Emissivity method, robust regression, high-temperature measurements

## Abstract

Accurate temperature and emissivity separation from thermal infrared radiance is essential for characterizing materials under high-temperature laboratory conditions. Existing temperature and emissivity separation methods have largely been developed for multispectral remote sensing applications, where long atmospheric path lengths require extensive atmospheric compensation. In contrast, the current work considers hyperspectral laboratory measurements acquired over a short optical path, where atmospheric effects are comparatively small but increased measurement uncertainty remains within portions of the measured spectrum. The ABB MR304 FTIR spectrometer used in this study exhibits reduced optical transmission below approximately 2.5 μm, producing increased measurement uncertainty within the spectral region containing much of the temperature information. To address these conditions, a modified Gray Body Emissivity method, referred to as the Robust Ratio Linear Fitting method, was developed using robust linear regression, spectral masking, and iterative temperature refinement. The algorithm was validated by comparing the retrieved temperatures with pyrometer measurements and the retrieved spectral emissivities with a high-accuracy spectral emissivity database collected using a SOC-100 hemispherical directional reflectometer. When applied to radiance measurements of a carbon phenolic sample heated using a plasma torch and measured with an ABB MR304 FTIR spectrometer, the algorithm retrieved temperatures with a mean absolute percentage error of 3.05% and spectral emissivities with a mean absolute percentage error of 3.13% relative to the SOC-100 reference measurements. Although the method is ineffective at lower temperatures where the peak of the Planck radiance lies within excluded spectral regions, the results demonstrate that the proposed approach provides accurate temperature and emissivity retrieval for high-temperature laboratory FTIR measurements acquired under these experimental conditions.

## 1. Introduction

### 1.1. Motivation

The ability to characterize materials using infrared (IR) spectral measurements has been an important area of research for several decades. Many approaches rely on accurate laboratory databases of spectral emissivity to understand the radiative properties of materials and, ultimately, support material characterization and identification. One challenge in developing these databases is that spectral emissivity can vary with temperature, requiring accurate measurements over a wide range of temperatures.

Plasma torches rapidly heat materials to temperatures approaching 2500 K, where direct spectral emissivity measurements are no longer practical. Similarly, extreme aerodynamic heating can produce substantial temperature increases that alter the spectral emissivity of a material. Characterizing these temperature-dependent changes is therefore important for understanding material behavior under extreme thermal conditions.

One of the first steps in processing thermal infrared radiometric data is separating the measured radiance into its temperature and spectral emissivity components. Accurate temperature and emissivity separation is essential because spectral emissivity is the intrinsic material property used for comparison with laboratory reference databases, while the recovered temperature provides important information regarding the thermal state of the material. Although numerous temperature and emissivity separation methods have been developed for multispectral and hyperspectral remote sensing applications, many are designed for long optical path lengths where atmospheric compensation is a primary concern. High-temperature laboratory hyperspectral measurements present a different set of challenges, motivating the development of approaches tailored to these experimental conditions.

### 1.2. Prior Research

To accurately separate temperature and emissivity from radiometric data, numerous temperature and emissivity separation techniques have been developed over the past four decades. These methods have been applied to a wide range of remote sensing applications, including environmental monitoring, geology, and the characterization of natural and engineered materials. Accurate retrieval of both temperature and spectral emissivity is essential for understanding the thermal behavior and radiative properties of natural and engineered materials. Extracting physical temperature from measured radiance requires knowledge of the surface emissivity at the observed wavelengths. The fundamental challenge is that hyperspectral measurements provide *N* radiance observations but contain N+1 unknowns, consisting of the surface temperature and the emissivity at each wavelength. This ill-posed inverse problem has motivated the development of numerous temperature and emissivity separation algorithms, each relying on different physical assumptions and retrieval strategies.

Temperature and emissivity separation methods have continued to evolve over the past several decades. One of the earliest approaches was the Channel 6 emittance model developed by Kahle et al. [1], which estimates land surface temperature by assuming a relatively constant emissivity within a thermal infrared reference band before iteratively refining emissivity estimates at other wavelengths. This method was later applied to map alluvial fans in Death Valley, California, using NASA’s 8–12 μm six-channel airborne Thermal Infrared Multispectral Scanner (TIMS) [2]. More advanced multispectral approaches were subsequently developed, including the Split-Window method [3] and the Normalization Emissivity Method (NEM) [4], both of which improve temperature retrieval by incorporating atmospheric compensation and additional spectral information. More recently, machine learning has been incorporated into traditional temperature and emissivity separation algorithms. For example, Cheng et al. [5] combined a knowledge-guided neural network with the Split-Window algorithm to improve land surface temperature retrieval for satellite-based multispectral observations.

Although these multispectral methods have demonstrated excellent performance for satellite and airborne remote sensing applications, they were developed primarily for multispectral imaging systems that acquire a small number of thermal infrared bands and require significant atmospheric compensation over long optical path lengths. In contrast, the ABB MR304 FTIR (ABB Measurement and Analytics, Zurich, Switzerland) used in this study acquires hyperspectral radiance measurements consisting of more than 9000 spectral samples over a short laboratory optical path where atmospheric effects are comparatively small. Consequently, the assumptions and design objectives of many existing multispectral temperature and emissivity separation methods are not well matched to the experimental conditions considered in this work.

Because the Linear Spectral Emissivity Constraint Method (LSEC) was specifically developed for hyperspectral thermal infrared measurements, it provides a more appropriate comparison for the laboratory FTIR data considered in this work than multispectral temperature and emissivity separation algorithms [6]. However, like many existing hyperspectral retrieval methods, it relies on accurate radiance measurements across the spectral region most sensitive to temperature. As shown later in this work, the reduced optical transmission of the MR304 FTIR below approximately 2.5 μm presents challenges for this approach when applied to the high-temperature laboratory measurements considered in this study.

These differences motivate the development of temperature and emissivity separation methods designed for laboratory measurements, where the optical path length is short but residual atmospheric absorption can still influence portions of the measured spectrum. To address this, the current work presents the Robust Ratio Linear Fitting (RRF) method, a modified implementation of the Gray Body Emissivity (GBE) method [7] developed for the experimental conditions of this study. The RRF method incorporates robust linear regression, spectral masking, and an iterative temperature refinement procedure to improve the stability of the temperature retrieval when residual atmospheric absorption and other spectral artifacts remain present. The GBE method, which forms the foundation of the RRF algorithm, is described in detail in Section 2.2.

### 1.3. Approach of Current Work

The first objective of this research was to collect a database of spectral emissivities for a given sample using the SOC-100 (Surface Optics Corporation, San Diego, CA, USA) at the Air Force Institute of Technology (AFIT), allowing the spectral features of the sample to be characterized.

Another objective of this research was to determine how the sample’s spectral emissivity changed as its temperature increased during plasma heating. Since the SOC-100 is only capable of collecting spectral emissivity measurements up to approximately 800 K, measurements at higher temperatures were collected at the Plasmatron X facility (University of Illinois Urbana-Champaign, Champaign, IL, USA). These measurements were obtained using the MR304 FTIR as it viewed the sample through an observation port while the sample was heated by a plasma torch to temperatures exceeding 2500 K.

The final objective of this research was to develop and evaluate a new temperature and emissivity separation technique called the RRF method. The RRF method incorporates characteristics of the Gray Body method while providing a temperature and emissivity separation approach developed for the experimental conditions of this study. The resulting temperature and spectral emissivity estimates were compared with reference spectral emissivity measurements from the SOC-100 and temperature measurements from a pair of pyrometers collected during the UIUC campaign. These comparisons were used to evaluate the accuracy and limitations of the RRF method for a high-temperature carbon phenolic sample.

### 1.4. Overview

The remainder of this paper is organized as follows. Section 2 describes the experimental setup, including the SOC-100 hemispherical directional reflectometer, the MR304 FTIR spectrometer, and the high-temperature plasma torch experiment. Section 3 presents the Gray Body Emissivity method and the proposed Robust Ratio Linear Fitting method for temperature and emissivity separation. Section 4 presents the experimental results, including comparisons between the Robust Ratio Linear Fitting method, the Linear Spectral Emissivity Constraint method, pyrometer temperature measurements, and the available SOC-100 spectral emissivity reference data. Section 5 and Section 6 present the conclusions and future work.

## 2. Data Collection

This section describes the data used throughout this research, including the highly accurate spectral emissivity measurements collected using the SOC-100 hemispherical directional reflectometer and the high-temperature radiometric measurements collected at the University of Illinois Urbana-Champaign (UIUC). The experimental configuration, including the ABB MR304 FTIR spectrometer, plasma torch facility, and supporting instrumentation, is also described. Although high-temperature measurements were collected for multiple materials during the plasma torch experiments, this work focuses on the carbon phenolic sample to evaluate the RRF method under the experimental conditions considered in this study. A more comprehensive comparison of the RRF method across multiple materials will be presented in future work.

### 2.1. SOC-100 Spectral Emissivity Database

The SOC-100, developed by Surface Optics Corporation, is an instrument designed to measure the hemispherical directional reflectance (HDR) of materials as a function of wavelength and incident angle. The instrument measures spectral reflectance as a function of polarization, temperature, and viewing geometry while accounting for both diffuse and specular reflection components.

**Figure 1:**
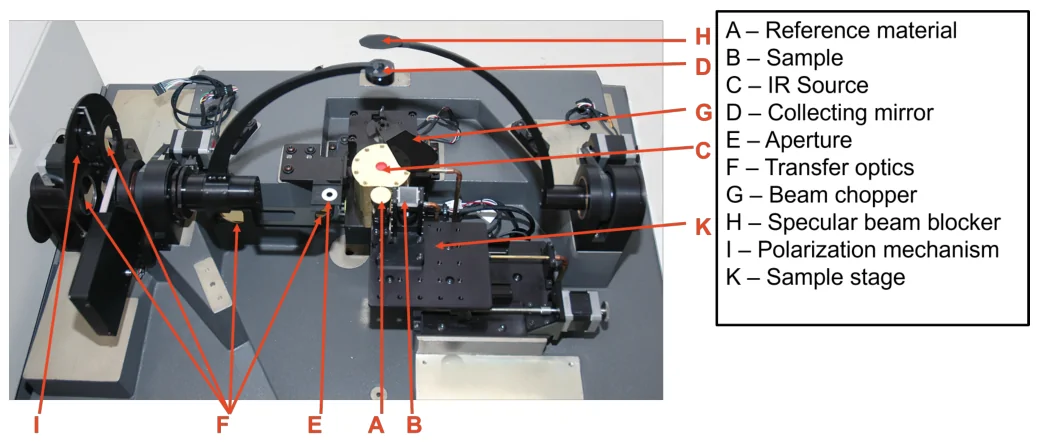
Internal components of the SOC-100 used to measure hemispherical directional reflectance (HDR). The gold hemiellipsoid has been removed to expose the optical components [8].

The instrument employs a Nicolet iS50 FTIR in conjunction with a gold-plated hemiellipsoid reflecting surface to measure spectral reflectance. An infrared source located at one focus of the hemiellipsoid illuminates the gold-plated reflecting surface, which redirects the radiation toward the second focus where either the sample or a gold reference is positioned. The light reflects off the sample or reference onto a collecting mirror, set at a user-specified angle, which then directs the light into the FTIR after passing through a polarization mechanism. The internal optical arrangement is depicted in Figure 1. This figure shows the system with the gold hemiellipsoid removed to expose the underlying optics.

The SOC-100 was configured with a KBr beam splitter, a DTGS detector, and a KBr detector window, providing a spectral range from 1.5 µm to 25 µm [8]. The instrument is capable of measuring spectral reflectance at incident angles ranging from 8° to 80° relative to the surface normal.

To measure HDR, the SOC-100 collects four separate readings: two from the sample (with the IR source covered and uncovered) and two from the gold reference. The covered measurement acts as a background measurement which is subtracted from the uncovered measurement. The four measurements are processed to determine the HDR using:

(1) $$\rho_{\mathrm{Sample}}\left(\lambda \right)=\frac{L_{\text{Sample-Uncov}}\left(\lambda \right)-L_{\text{Sample-Cov}}\left(\lambda \right)}{L_{\text{Ref-Uncov}}\left(\lambda \right)-L_{\text{Ref-Cov}}\left(\lambda \right)}\rho_{\mathrm{Ref}}\left(\lambda \right),$$

where *L* represents the radiance for the sample or reference in covered and uncovered states. The directional emissivity, ϵ(λ,θ), is then calculated as:

(2) $$\epsilon \left(\lambda ,\theta \right)=1-\rho_{\mathrm{Sample}}\left(\lambda ,\theta \right).$$

Spectral emissivity measurements were collected for the carbon phenolic sample investigated throughout this work. The sample had a weight of 5.378 g and a thickness of 6.52 mm. Data was acquired for both parallel and perpendicular polarization settings. For each polarization, measurements were taken at incident zenith angles from 10° to 80° in increments of 2°, at a single azimuthal angle of 0°. In total, 36 angular measurements were taken per polarization. Additional measurements were performed using a heated sample holder to investigate temperature-dependent changes in spectral emissivity. The analysis presented in this work focuses on the 2–14 μm wavelength range, corresponding to the spectral region used for the high-temperature MR304 FTIR measurements at UIUC. All SOC-100 measurements were acquired at a spectral resolution of 16 cm^−1^. The resulting database serves as the reference spectral emissivity dataset used to evaluate the temperature and emissivity separation results presented later in this paper.

### 2.2. ABB MR304 FTIR Data Collection at UIUC

**Figure 2:**
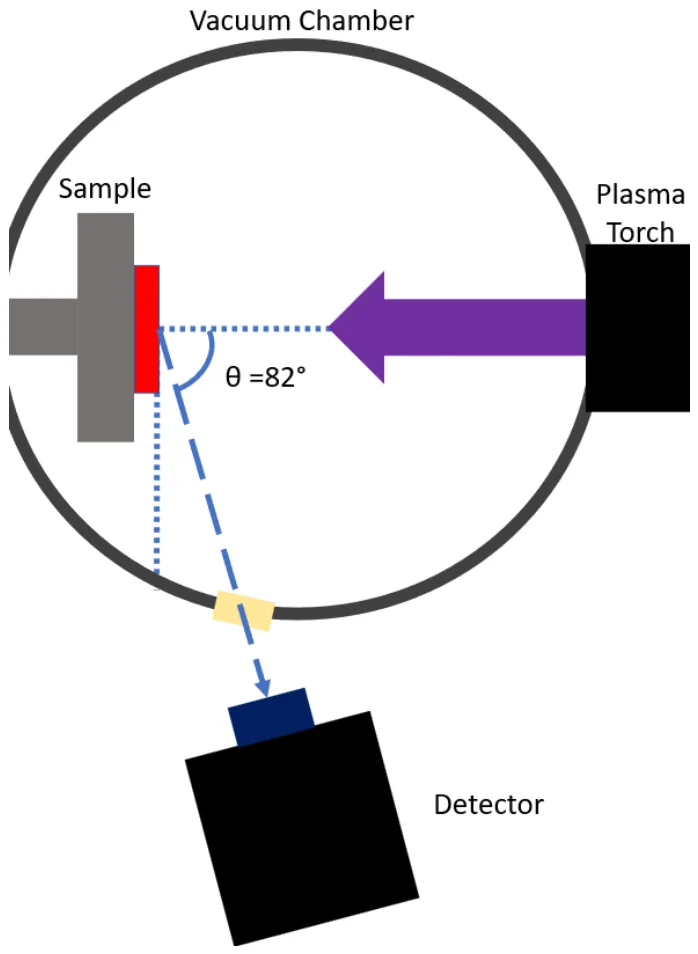
Schematic of the experimental setup used to collect spectral radiance measurements with the MR304 FTIR. The instrument viewed the sample through an observation window while the sample was heated using a plasma torch. The observation window transmits radiation over the approximate 2–12 μm spectral range used by the MR304 FTIR.

The sample was heated to temperatures exceeding 2500 K using a plasma torch. The plasma torch operated within a reduced-pressure chamber to protect its components and reduce atmospheric interference within the measurement environment. Observation windows installed on the chamber provided optical access for the instruments. The available optical port at the Plasmatron facility limited the MR304 FTIR viewing geometry to approximately 82° relative to the surface normal. A schematic of the experimental setup is shown in Figure 2.

High-temperature data were collected using the MR304 FTIR and two infrared pyrometers. The MR304 FTIR measured the spectral radiance emitted by the sample through the chamber window, while the pyrometers provided independent temperature measurements throughout the heating process. A Fluke E2RL-F1-L-0-0 pyrometer (Fluke Corporation, Everett, WA, USA) was used over a temperature range of approximately 250 °C to 1200 °C and operated using a nominal one/two-color measurement near 1.6 μm. A Heitronics K18.03RD pyrometer (HEITRONICS Infrarot Messtechnik GmbH, Wiesbaden, Germany) was used over a temperature range of approximately 1000 °C to 3000 °C and operated over the 0.85–1.10 μm and 0.95–1.10 μm spectral bands. The Fluke pyrometer was primarily used during the initial stages of heating, while the Heitronics pyrometer was used after the sample reached higher temperatures.

The FTIR and pyrometer measurements were acquired independently and synchronized during post-processing. A stabilization point was identified within each dataset, and a constant time offset was applied to align the FTIR and pyrometer measurements. Following temporal alignment, the Heitronics pyrometer temperatures were linearly interpolated to the FTIR measurement times. The resulting pyrometer temperatures served as the reference measurements used to evaluate the accuracy and limitations of the temperature retrievals.

**Figure 3:**
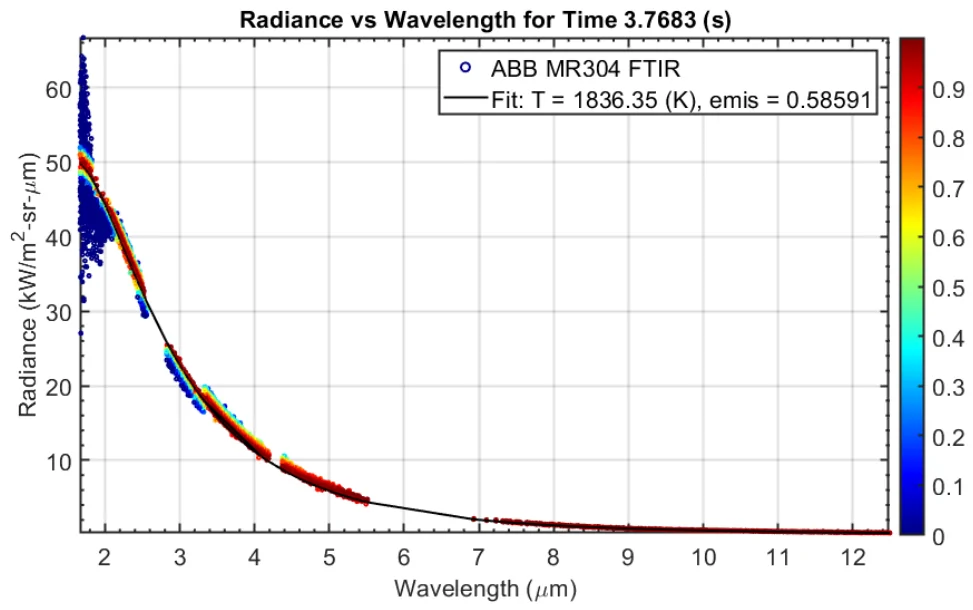
Example of a measured FTIR radiance spectrum illustrating the increased measurement uncertainty observed below approximately 2.5 μm. The reduced optical transmission of the MR304 FTIR within this wavelength range results in a lower signal-to-noise ratio than at longer wavelengths. This figure is taken from the RRF analysis and is included only to illustrate the measurement characteristics of the MR304 FTIR.

The MR304 FTIR measures spectral radiance over a wavelength range of approximately 2–15 μm. However, the instrument exhibits reduced optical transmission below approximately 2.5 μm, resulting in a lower signal-to-noise ratio within this portion of the measured spectrum. This limitation is particularly important for the high-temperature measurements considered in this work because a substantial portion of the emitted radiance occurs at shorter wavelengths. An example of the increased measurement uncertainty observed within this wavelength range is shown in Figure 3.

Prior to analysis, the measured spectra were radiometrically calibrated using blackbody sources maintained at approximately 500 °C, 1000 °C, and 1250 °C. Since the sample temperatures exceeded the highest calibration temperature, a three-point linear extrapolation was used to extend the calibration from approximately 1250 °C to 2500 °C. Previous work by Crego et al. evaluated a similar calibration approach using blackbody temperatures of 400 °C, 500 °C, and 600 °C to extrapolate the calibration to approximately 1200 °C. For the high-intensity measurements considered in that study, the three-point linear extrapolation produced sum of squared residual values ranging from 2.27 to 43.35 $\times \;10^{-9}\;W^{2}\;{\mathrm{cm}}^{-4}\;{\mathrm{sr}}^{-2}\;{\mathrm{cm}}^{-2}$ [9]. Although the calibration temperatures used in the present work differ, the same three-point linear extrapolation approach was applied.

## 3. Methodology

### 3.1. Gray Body Emissivity Method

The Gray Body Emissivity (GBE) method assumes that spectral emissivity varies slowly with wavelength and can therefore be approximated as constant over the spectral range of interest. Under this gray body assumption, the temperature and emissivity of a target can be estimated simultaneously from measured spectral radiance [7]. The mathematical foundation of the GBE method begins with the radiance equation,

(3) L(λ)=ϵ(λ)B(λ,T),

where L(λ) is the measured spectral radiance at wavelength λ, ϵ(λ) is the spectral emissivity, and B(λ,T) is the Planck function,

(4) $$B\left(\lambda ,T\right)=\frac{2hc^{2}}{\lambda^{5}}\frac{1}{\exp\left(\frac{hc}{\lambda kT}\right)-1}.$$

The GBE method simplifies Equation (3) by assuming that emissivity is independent of wavelength,

(5) ϵ(λ)=ϵ,

which reduces Equation (3) to

(6) L(λ)=ϵB(λ,T).

Under this assumption, the surface temperature and gray body emissivity are estimated by minimizing the difference between the measured spectral radiance and the modeled gray body radiance. The objective function is defined as

(7) $$E=\sum_{i=1}^{N}{\left[L\left(\lambda_{i}\right)-\epsilon B\left(\lambda_{i},T\right)\right]}^{2},$$

where *N* is the number of wavelength samples included in the fit.

For a given temperature, the gray body emissivity is obtained by minimizing Equation (7) with respect to ϵ. Setting the first derivative equal to zero gives

(8) $$\frac{\partial E}{\partial \epsilon }=-2\sum_{i=1}^{N}B\left(\lambda_{i},T\right)\left[L\left(\lambda_{i}\right)-\epsilon B\left(\lambda_{i},T\right)\right]=0.$$

Rearranging this expression yields the least-squares estimate of the gray body emissivity,

(9) $$\epsilon =\frac{\sum L\left(\lambda_{i}\right)B\left(\lambda_{i},T\right)}{\sum B^{2}\left(\lambda_{i},T\right)}.$$

The temperature and gray body emissivity are determined iteratively by estimating the gray body emissivity for a candidate temperature, evaluating the objective function, and repeating the process until the minimum error is obtained. This process provides the gray body temperature and emissivity that best reproduce the measured spectral radiance.

The gray body assumption provides a useful approximation for the carbon phenolic sample investigated in this work and serves as the physical foundation for the Robust Ratio Linear Fitting method. Rather than modifying the underlying gray body model, the RRF method modifies the parameter estimation procedure by incorporating robust linear regression, spectral masking, and iterative temperature refinement to improve the stability of the temperature and emissivity retrieval for the high-temperature laboratory FTIR measurements considered in this study.

### 3.2. Robust Ratio Linear Fitting Method and Algorithm

The following section describes a new method of temperature and emissivity separation called the Robust Ratio Linear Fitting Method. This section includes an explanation of how the algorithm functions and the reasons for its design.

The Robust Ratio Linear Fitting method does not follow the previously described GBE method exactly, but instead incorporates aspects of the GBE method into an approach developed for the experimental conditions of this study. It leverages the “robustfit” function from MATLAB (R2023a) to perform a weighted gray body fit on radiance data obtained from the FTIR. In this implementation, the robust regression model was defined as a linear relationship between the measured FTIR spectral radiance and the calculated Planck radiance at a candidate temperature. Specifically, the measured radiance was treated as the dependent variable, while the corresponding Planck radiance was treated as the independent variable in the robust linear regression. The resulting slope coefficient from the regression represents the estimated gray body emissivity, while the intercept accounts for any residual offset in the radiance measurements.

The MATLAB robustfit function performs this regression using an iteratively reweighted least-squares approach, where individual wavelength samples are assigned weights based on their contribution to the regression residuals. The default bisquare weighting function was used to reduce the influence of spectral outliers while preserving valid spectral information. Although the measurements were collected in a laboratory environment with a relatively short optical path length, residual atmospheric absorption and other spectral artifacts remain present within portions of the measured spectrum. In addition, the MR304 FTIR exhibits reduced optical transmission below approximately 2.5 μm, producing a lower signal-to-noise ratio within the wavelength region containing much of the thermal emission for the high-temperature measurements considered in this work. Rather than discarding these wavelength samples, the robust regression assigns lower weights to samples with larger residuals, allowing the information contained within these measurements to contribute to the temperature optimization while reducing their influence on the final solution.

Before each regression was performed, atmospheric absorption bands and other unreliable spectral regions were removed using the previously defined spectral mask. Specifically, the atmospheric absorption bands at 2.54–2.82 μm, 4.19–4.37 μm, and 5.51–6.93 μm were excluded from the regression and were not interpolated or otherwise reconstructed. The same spectral mask was applied during each robust regression iteration to ensure that wavelengths affected by atmospheric absorption did not influence the temperature optimization process. The remaining wavelength intervals were used as inputs to the robust fitting procedure.

The temperature search was constrained between 250 K and 3000 K, and successive refinement was performed using temperature increments of 100 K, 10 K, 1 K, 0.1 K, and 0.01 K. The smaller temperature increments were selected to ensure that the temperature step size did not become a limiting factor in the algorithm performance. Although this refinement exceeds the practical measurement accuracy of the experimental data, computational efficiency was not the primary objective during the initial development of the algorithm. At each tested temperature, the robust regression was performed and the resulting median absolute deviation of the regression residuals ($\sigma_{MAD}$) was used as the fitting metric. The temperature corresponding to the minimum $\sigma_{MAD}$ value was selected as the optimal temperature. A physical constraint was applied to the fitted gray body emissivity by accepting only physically possible emissivity values (0 < ϵ < 1). Solutions that produced emissivity values outside this range were rejected.

The algorithm combines robust linear regression, spectral masking, and an iterative temperature search to determine the temperature that best reproduces the measured radiance. By reducing the influence of spectral outliers during the temperature optimization process, the algorithm provides more reliable temperature and emissivity estimates, especially when residual atmospheric absorption and other spectral artifacts remain present in the measured data.

The procedure outlined above is described in detail below. The first step of the RRF algorithm is to estimate the sample temperature. Rather than solving directly for temperature and emissivity simultaneously, the algorithm evaluates a series of candidate temperatures. For each candidate temperature, the corresponding blackbody radiance is first calculated using Planck’s Law (Equation (4)), and the agreement between the measured FTIR radiance and the calculated gray body radiance is evaluated through a robust linear regression.

For each candidate temperature, MATLAB’s robustfit function solves the weighted least-squares problem

(10) $$\min_{\epsilon ,b_{0}}\sum_{i=1}^{n}w_{i}{\left[L\left(\lambda_{i}\right)-\left(\epsilon B\left(\lambda_{i},T\right)+b_{0}\right)\right]}^{2},$$

where $w_{i}$ are the robust weights assigned to each wavelength sample, $L(\lambda_{i})$ is the measured spectral radiance, $B(\lambda_{i},T)$ is the calculated Planck radiance at the candidate temperature, ϵ is the gray body emissivity represented by the regression slope, $b_{0}$ is the fitted regression intercept, and *n* is the number of wavelength samples included in the regression. Unlike the maximum likelihood formulation used in the original GBE method [7], the RRF method uses this robust weighted least-squares formulation to improve the stability of the gray body fit under the experimental conditions considered in this study.

This weighted least-squares formulation was selected because it directly minimizes the difference between the measured FTIR radiance and the gray body radiance predicted by Planck’s Law for a given candidate temperature. Unlike an ordinary least-squares regression, the robust regression assigns lower weights to wavelength samples with larger residuals using an iteratively reweighted least-squares procedure. This reduces the influence of spectral samples affected by increased measurement uncertainty while preserving the remaining spectral information during the temperature optimization process.

The resulting robust fit weights are represented by the colorbar, where spectral samples containing larger residual errors receive lower weights during the regression process.

**Figure 4:**
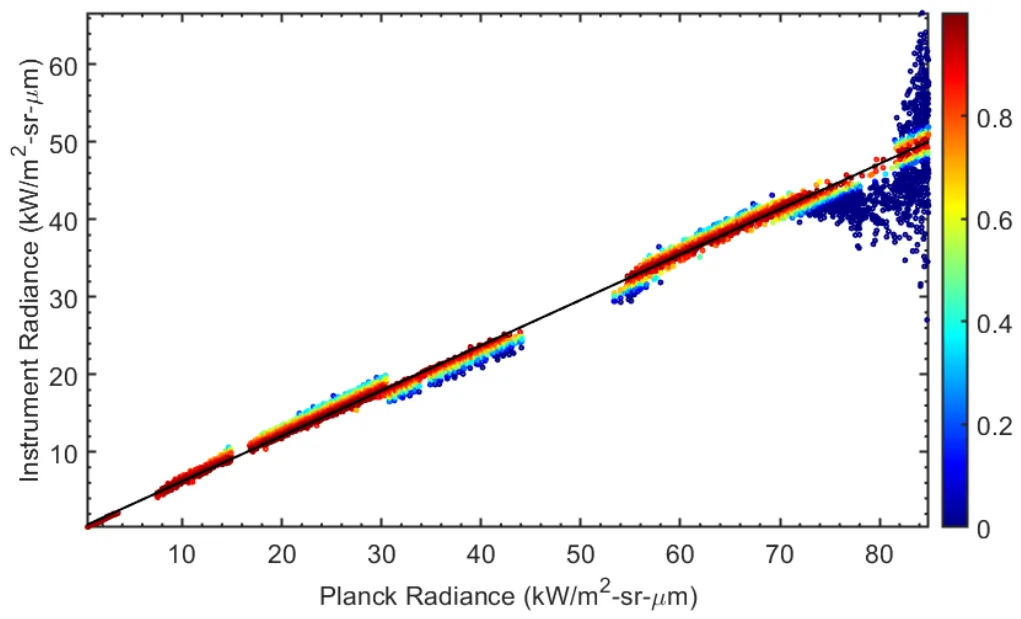
The gray body Planck fit is plotted against the radiance from the FTIR in this figure to demonstrate the robust linear regression between the measured FTIR radiance and the calculated Planck radiance. The robust regression reduces the influence of spectral outliers by assigning lower weights to data points with larger residual errors. It should be noted that the colorbar indicates the weights assigned to each data point.

The slope of the robust linear fit in Figure 4 represents the gray body emissivity of the sample at the tested temperature. Once the robust regression has been completed, the residual error at each wavelength is calculated as

(11) $$r_{i}=L\left(\lambda_{i}\right)-\left(\epsilon B\left(\lambda_{i},T\right)+b_{0}\right),$$

where $r_{i}$ represents the difference between the measured spectral radiance and the fitted gray body radiance. The overall quality of the gray body fit for the candidate temperature is evaluated using the median absolute deviation of the regression residuals,

(12) $$\sigma_{MAD}=\mathrm{median}\left(\left|r_{i}-\mathrm{median}\left(r_{i}\right)\right|\right),$$

where smaller values of $\sigma_{MAD}$ indicate a closer agreement between the measured FTIR radiance and the fitted gray body radiance.

**Figure 5:**
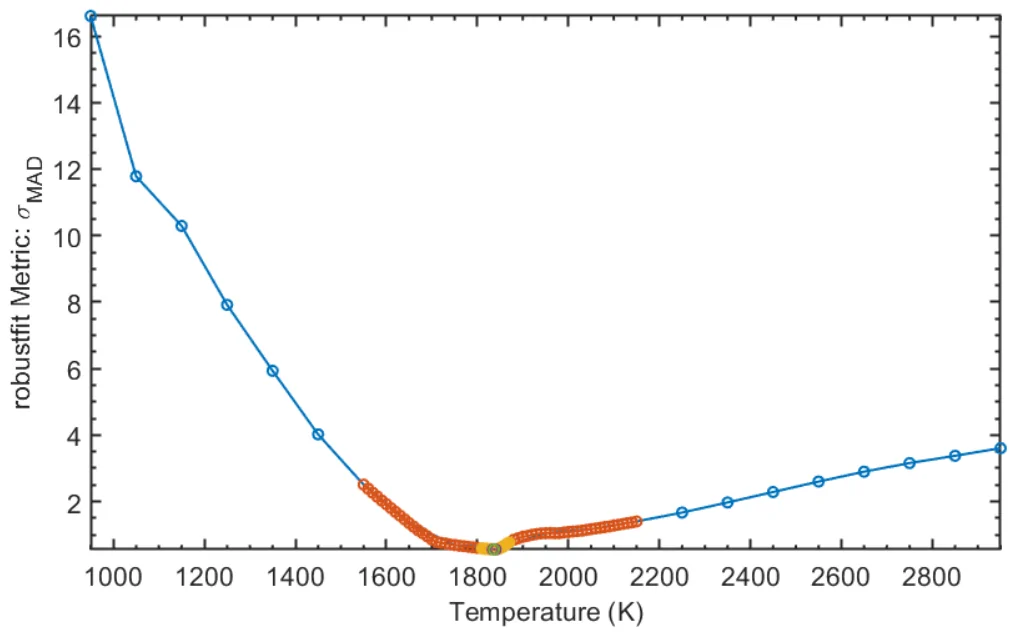
This figure illustrates the successive temperature refinement procedure used by the RRF algorithm. The blue data points indicate tested temperatures at every 100 K and the minimum error from these temperatures. The orange data points are tested temperatures at every 10 K followed by yellow data points every 1 K, purple data points every 0.1 K and green data points every 0.01 K.

The robust regression and calculation of $\sigma_{MAD}$ are repeated for each candidate temperature within the search range. The candidate temperature corresponding to the minimum $\sigma_{MAD}$ value is selected as the optimal temperature. The successive temperature refinement described previously is then performed using progressively smaller temperature increments of 100 K, 10 K, 1 K, 0.1 K, and 0.01 K until the final temperature resolution is reached, as illustrated in Figure 5.

**Figure 6:**
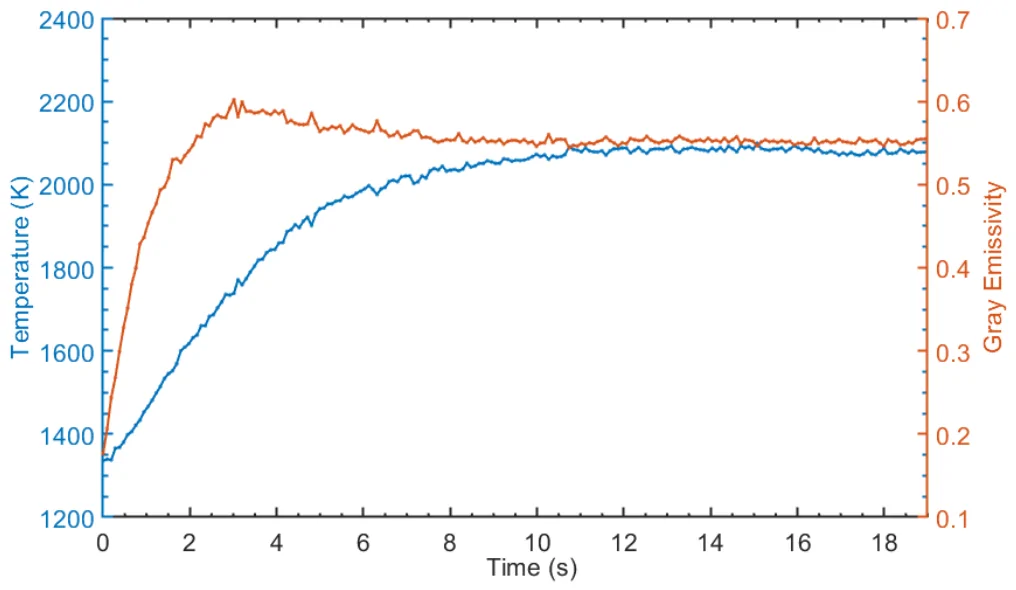
This figure shows the rapid rise in temperature the sample underwent during heating before reaching a steady temperature state after 12 s of collection. The figure also shows the corresponding gray body emissivity, which reaches a steady state after the sample temperature stabilizes.

These steps are repeated at each time interval and an array of temperatures and corresponding gray body emissivities is built up. The accumulation of these temperatures and gray body emissivities for the sample is shown in Figure 6.

**Figure 7:**
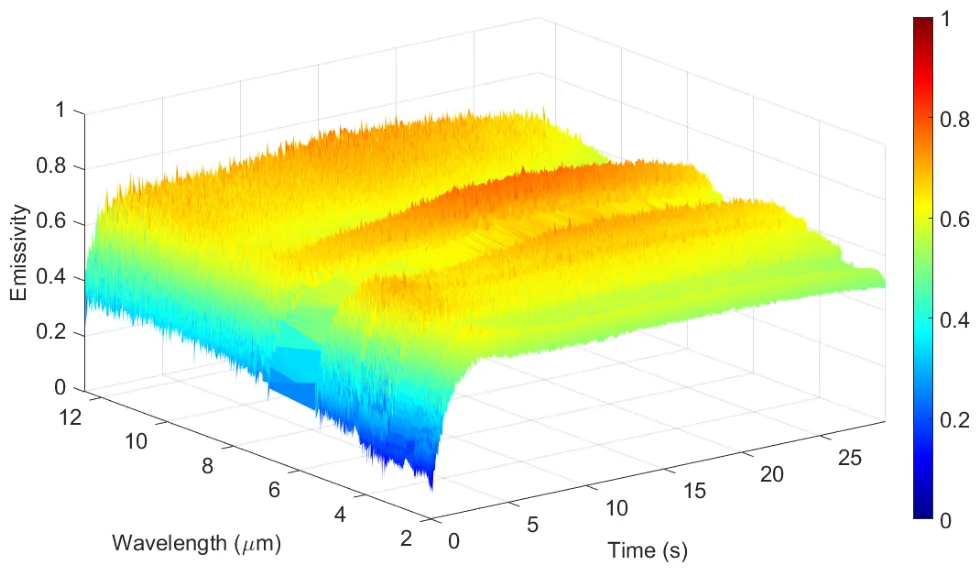
This figure shows an example of the spectral emissivity determined by the RRF algorithm when applied to MR304 FTIR data collected while the sample was heated by a plasma torch. The colorbar in all 3D plots throughout this paper represents emissivity.

The data used to demonstrate the algorithm were collected during the UIUC campaign, where the sample was heated to temperatures that typically stabilized between approximately 2100 K and 2600 K. Furthermore, once the temperature at each time interval is determined, this value is substituted into Planck’s equation to determine the spectral emissivity at each time interval as shown in Figure 7. Section 4 will present a direct comparison of these derived temperature values with those from the pyrometer, alongside a comparison of the high-temperature spectral emissivity with highly accurate SOC-100 measurements to evaluate their consistency.

The spectral emissivity ϵ(λ,t) at wavelength λ and time *t* is calculated as:

(13) $$\epsilon \left(\lambda ,t\right)=\frac{L(\lambda ,t)}{B(\lambda ,T(t))},$$

where L(λ,t) is the measured radiance at wavelength λ and time *t*, and T(t) is the temperature determined by the RRF algorithm at time *t*.

The RRF method was developed specifically for the experimental conditions considered in this study and evaluated using high-temperature MR304 FTIR measurements collected during the UIUC campaign. Although the laboratory measurements were acquired over a relatively short optical path length, the reduced optical transmission of the MR304 FTIR below approximately 2.5 μm produces increased measurement uncertainty within the portion of the spectrum containing much of the temperature information. Residual atmospheric absorption also remains present within several isolated wavelength regions. By combining spectral masking with robust regression weighting, the RRF method reduces the influence of these measurements while still utilizing the remaining spectral information during the temperature optimization process. It should be noted that Appendix A includes an uncertainty analysis for the retrieved temperatures and spectral emissivities in this study. Section 4 evaluates the accuracy of the method through comparisons with pyrometer temperature measurements and SOC-100 spectral emissivity measurements.

## 4. Results

This section presents the experimental results obtained throughout this study. It begins with the SOC-100 spectral emissivity measurements for the carbon phenolic sample, followed by the results obtained using the Linear Spectral Emissivity Constraint and Robust Ratio Linear Fitting methods. The retrieved temperatures and spectral emissivities are compared with the available pyrometer and SOC-100 reference measurements to evaluate the performance of each approach.

### 4.1. SOC-100 Measurements

The SOC-100 measurements were used to construct a spectral emissivity database for the carbon phenolic sample. Measurements were acquired over a range of incident zenith angles and polarization states to characterize the angular dependence of the sample’s spectral emissivity. Unless otherwise noted, figures displaying spectral emissivity as a function of incident angle use a consistent color scale, where red corresponds to the smallest incident zenith angle (10°) and purple corresponds to the largest incident zenith angle (80°). Measurements were collected at 2° increments, resulting in 36 incident angles spanning 10° to 80°.

The SOC-100 records parallel and perpendicular polarization measurements, along with the average of the two polarizations. Because the averaged spectral emissivity was used as the reference dataset for evaluating the temperature and emissivity separation methods, the discussion below focuses primarily on the averaged polarization results.

**Figure 8:**
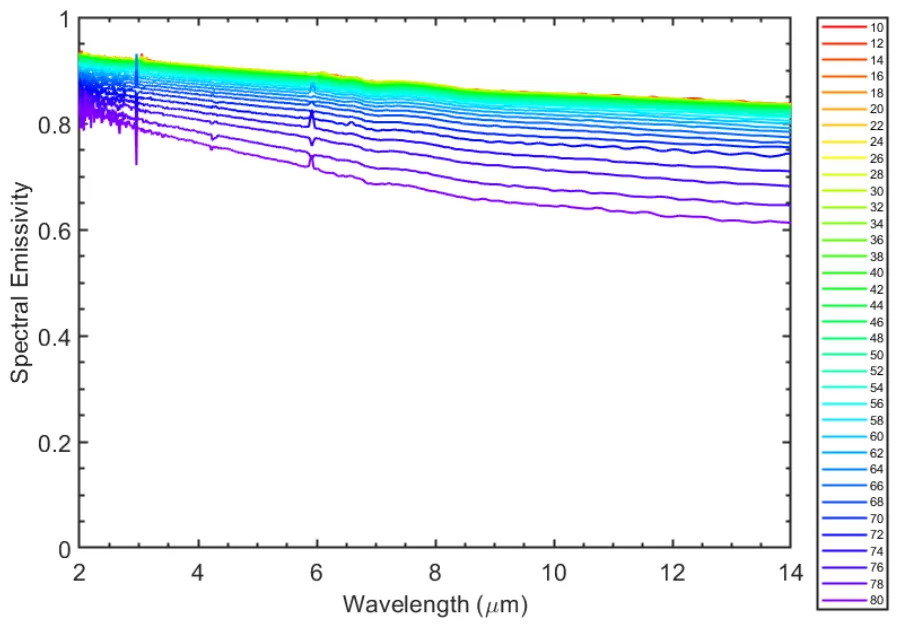
Average spectral emissivity of the carbon phenolic sample measured at incident zenith angles from 10° to 80°. The spectral emissivity remains relatively constant over most viewing angles, with increased angular dependence becoming apparent at larger zenith angles.

The averaged spectral emissivity remains relatively uniform across the measured wavelength range and exhibits only modest angular dependence over most incident angles, as shown in Figure 8.

**Figure 9:**
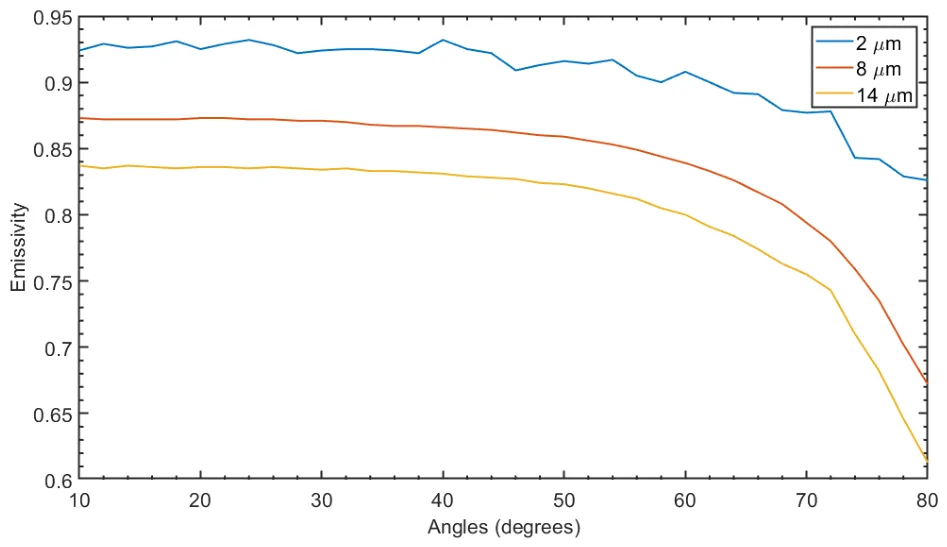
Angular dependence of the carbon phenolic sample at wavelengths of 2 μm, 8 μm, and 14 μm. The spectral emissivity remains nearly constant over much of the angular range before decreasing at larger incident zenith angles approaching grazing incidence.

Figure 9 illustrates the angular dependence of the sample at three representative wavelengths: 2 μm, 8 μm, and 14 μm. The spectral emissivity remains nearly constant for incident zenith angles below approximately 50°, after which a gradual decrease in emissivity is observed as the viewing geometry approaches grazing incidence.

The increased angular dependence observed near grazing incidence is particularly important because the MR304 FTIR viewed the sample at approximately 82°, slightly beyond the maximum measurement angle of the SOC-100. This behavior is considered when comparing the high-temperature FTIR-derived emissivities with the SOC-100 reference measurements in Section 4.2.

### 4.2. Temperature and Emissivity Separation Algorithms Results

This section presents the results obtained using the Linear Spectral Emissivity Constraint (LSEC) and Robust Ratio Linear Fitting (RRF) methods. The section also discusses the limitations associated with applying these temperature and emissivity separation methods to the high-temperature MR304 FTIR measurements collected at the Plasmatron facility. It should be noted that the zenith angle at which the FTIR examined the sample was near grazing, at approximately 82°. This viewing geometry was determined by the experimental configuration of the Plasmatron facility, as the available optical port to the sample was limited to this viewing angle. The SOC-100 is capable of measuring zenith angles up to 80° off normal to the sample surface, placing the FTIR viewing geometry slightly outside the measurement range of the instrument. As shown in Figure 9, the spectral emissivity of the sample exhibits increased angular dependence at higher zenith angles. To approximate the FTIR viewing geometry, the SOC-100 spectral emissivity at 82° was estimated by linearly extrapolating the angular trend observed between the measured 70° and 80° SOC-100 data. Although this extrapolation extends beyond the direct measurement range of the SOC-100 and introduces additional uncertainty, it is based on the measured angular behavior immediately preceding the extrapolation. Furthermore, the SOC-100 measurements were collected in 2° increments from 10° to 80°, providing a detailed characterization of the angular emissivity behavior and supporting a reasonable estimate of the emissivity at 82°. The extrapolated 82° spectral emissivity was then used as the reference for comparison with the FTIR-derived emissivity estimates. Consequently, while some uncertainty remains due to the angular extrapolation, the comparison provides a useful basis for evaluating the retrieved spectral emissivities produced by the LSEC and RRF methods.

The SOC-100 spectral emissivity measurements were collected at significantly lower temperatures than those encountered during the plasma heating experiment. The SOC-100 measurements represent the closest available reference for comparison because high-temperature spectral emissivity measurements for these materials are not currently available. As a result, the comparison assumes that the spectral emissivity measured at lower temperatures remains representative of the material at elevated temperatures. Although changes in emissivity may occur due to oxidation, structural degradation, or other temperature-dependent effects, evaluating these changes is beyond the scope of the present work and will require future high-temperature reference measurements.

The MR304 FTIR was unable to provide reliable measurements within the major atmospheric absorption bands at 2.54–2.82 μm, 4.19–4.37 μm, and 5.51–6.93 μm. These spectral regions were excluded from all subsequent analyses. In addition, the MR304 FTIR exhibits reduced optical transmission below approximately 2.5 μm, producing a lower signal-to-noise ratio within the portion of the spectrum containing much of the thermal emission from the high-temperature samples. Although these wavelengths were retained during the RRF analysis, their increased measurement uncertainty presents additional challenges for temperature and emissivity separation.

Excluding the atmospheric absorption bands, the FTIR provided measurements from 1.67–12.49 μm consisting of 9040 wavelength samples. Approximately 33 s of radiance data were collected during the heating experiment, corresponding to 352 measurement times. The SOC-100 spectral emissivity measurements span 2.00–14.09 μm with 558 wavelength samples. To enable direct comparison between the datasets, the SOC-100 spectral emissivity measurements were interpolated onto the FTIR wavelength grid using one-dimensional linear interpolation.

#### 4.2.1. Linear Spectral Emissivity Constraint Method Results

The LSEC method was applied to the FTIR data to provide a comparison with the proposed RRF method. The LSEC algorithm retrieves temperature and emissivity by modeling the spectral emissivity as a piecewise linear function and iteratively minimizing the difference between the measured and modeled radiance. Although the LSEC method was developed for hyperspectral temperature and emissivity separation, its performance depends on accurately resolving the portion of the measured spectrum containing the greatest temperature information.

The high temperatures encountered during the plasma heating experiment shift a substantial portion of the emitted radiance into the short-wavelength region below approximately 2.5 μm. Because the MR304 FTIR exhibits reduced optical transmission within this wavelength range, increased measurement uncertainty is introduced into the portion of the spectrum that contains much of the temperature information. Consequently, the retrieved temperature becomes particularly sensitive to measurement uncertainty within these wavelengths.

**Figure 10:**
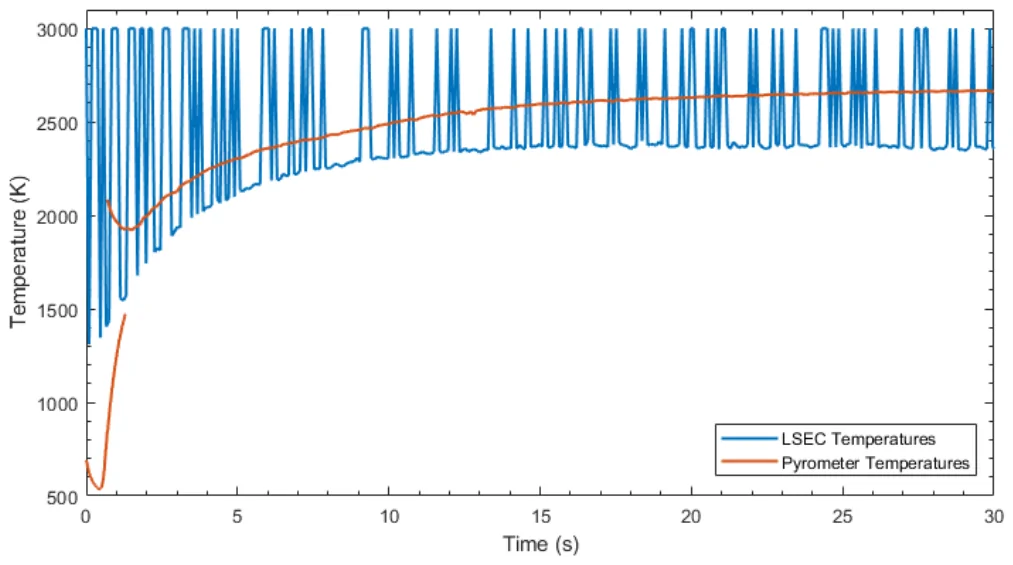
Comparison of temperatures retrieved using the LSEC method with the reference pyrometer measurements. Although agreement is observed during portions of the experiment, many LSEC retrievals converge to temperatures near the upper bound of the temperature search, indicating difficulty separating temperature and emissivity under the experimental conditions considered in this study.

The effect of this increased measurement uncertainty is evident in the temperatures retrieved by the LSEC method. Figure 10 compares the LSEC-derived temperatures with the reference pyrometer measurements collected during the plasma heating experiment. Although the LSEC method produces temperatures that agree with the pyrometer measurements during portions of the experiment, it frequently converges to temperatures substantially higher than those measured by the pyrometers, with many retrievals approaching the upper bound of the temperature search. Since the retrieved temperature directly influences the calculated spectral emissivity, these temperature errors propagate directly into the emissivity estimates.

**Figure 11:**
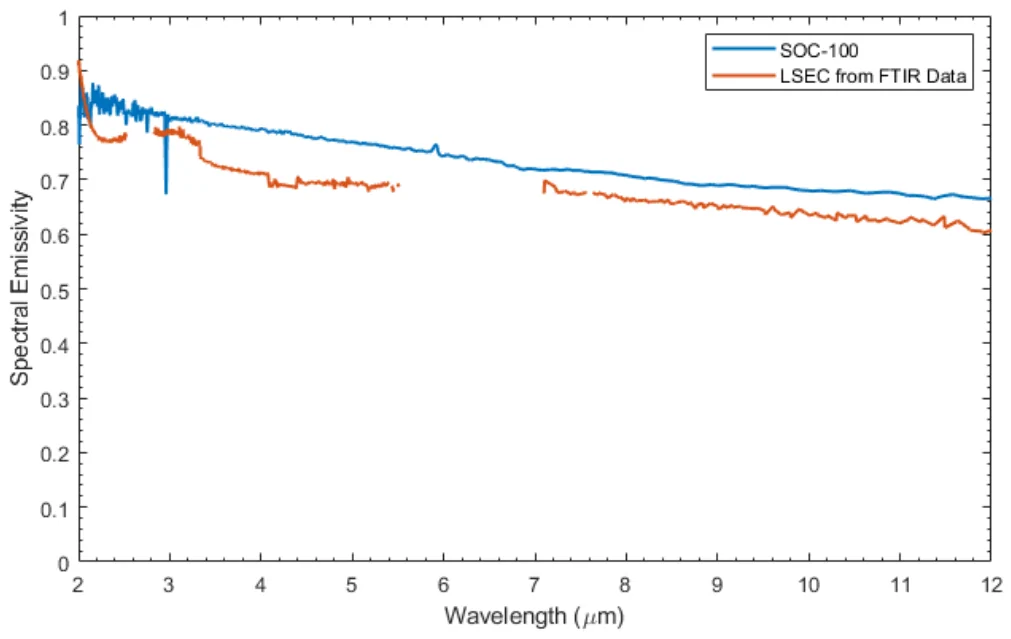
Representative example of a spectral emissivity retrieval obtained using the LSEC method for which the retrieved spectrum follows the overall trend of the SOC-100 reference measurements. Although the overall spectral trend follows the SOC-100 reference measurements, a noticeable offset remains across much of the measured spectrum. Gaps correspond to wavelength regions excluded from the analysis because reliable FTIR measurements were unavailable within the major atmospheric absorption bands.

Despite these limitations, the LSEC method produced spectral emissivity estimates that followed the general trend of the SOC-100 reference measurements for portions of the dataset. Figure 11 presents one such example. Although the retrieved spectral emissivity follows the general trend of the SOC-100 reference measurements, a noticeable offset remains across much of the measured spectrum. This result demonstrates that the LSEC method can reproduce the overall spectral behavior of the sample, but does not consistently recover the correct emissivity magnitude.

**Figure 12:**
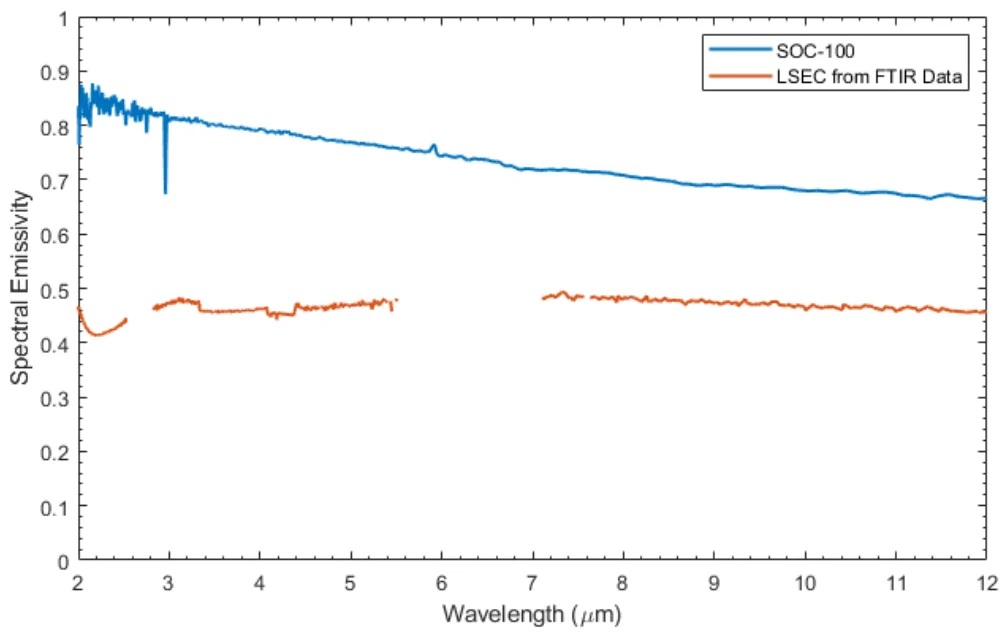
Representative example of a poor spectral emissivity retrieval obtained using the LSEC method. The overestimation of temperature results in spectral emissivity values that are consistently lower than the SOC-100 reference measurements across the measured wavelength range.

The Planck radiance is highly sensitive to temperature within the short-wavelength portion of the spectrum, causing errors in the measured radiance below approximately 2.5 μm to have a disproportionately large influence on the retrieved temperature. This sensitivity increases rapidly at shorter wavelengths, making temperature retrieval considerably more sensitive to measurement uncertainty in this portion of the spectrum than at longer wavelengths. As a result, even relatively small measurement uncertainties within this wavelength range can produce large errors in the estimated temperature, which then propagate directly into the retrieved spectral emissivity. Figure 12 shows an example in which the retrieved temperature converged to a value near the upper bound of the temperature search. The resulting overestimation of temperature produces spectral emissivity values that are consistently lower than the SOC-100 reference measurements across nearly the entire wavelength range. These results demonstrate the strong coupling between the retrieved temperature and emissivity and illustrate the sensitivity of the LSEC method to increased measurement uncertainty within the short-wavelength portion of the measured spectrum.

The results presented in this section demonstrate the challenges associated with applying existing temperature and emissivity separation methods to the high-temperature laboratory measurements considered in this study. In particular, increased measurement uncertainty within the short-wavelength portion of the MR304 FTIR spectrum complicates the temperature retrieval because a substantial fraction of the thermal emission occurs within this wavelength range. These observations motivated the development of the Robust Ratio Linear Fitting method, which retains the gray body formulation while modifying the parameter estimation procedure through robust regression, spectral masking, and iterative temperature refinement. Rather than discarding wavelength samples exhibiting increased measurement uncertainty, the RRF method reduces their influence during the temperature optimization while still utilizing the information they contain.

#### 4.2.2. Robust Ratio Linear Fitting Method Results

The RRF method was developed specifically for the experimental conditions considered in this study. Unlike the LSEC method, which exhibited difficulty retrieving accurate temperatures when increased measurement uncertainty was present within the wavelength range containing much of the temperature information, the RRF method retained stable temperature estimates throughout the high-temperature portion of the experiment by using robust regression during the temperature optimization. Rather than discarding wavelength samples exhibiting increased measurement uncertainty, the robust regression procedure automatically reduces their influence while still allowing those measurements to contribute to the solution.

**Figure 13:**
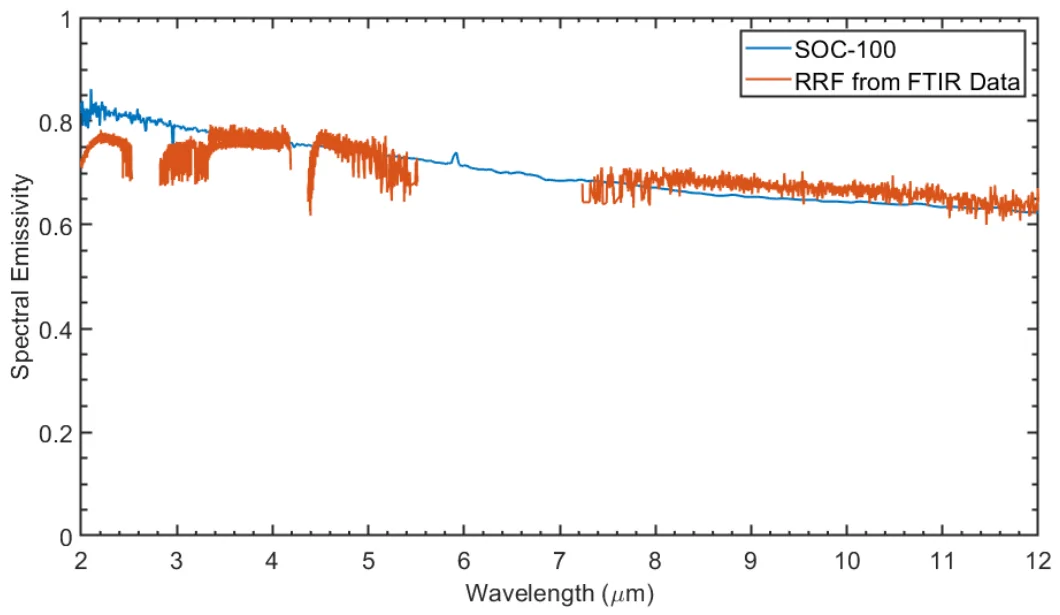
Comparison between the spectral emissivity retrieved using the RRF method and the SOC-100 reference measurements. The retrieved emissivity closely follows the overall spectral behavior of the SOC-100 data. Gaps correspond to wavelength regions excluded from the analysis because reliable FTIR measurements were unavailable within the major atmospheric absorption bands.

The retrieved spectral emissivity closely followed the trends observed in the SOC-100 reference measurements, although localized deviations were present across portions of the measured spectrum. Previous SOC-100 measurements collected between approximately 300 K and 700 K showed little change in the spectral emissivity of the carbon phenolic sample over that temperature range. Furthermore, Figure 13 suggests that the overall spectral behavior remains similar at temperatures exceeding 2000 K despite the remaining differences between the retrieved emissivity and the SOC-100 reference.

**Figure 14:**
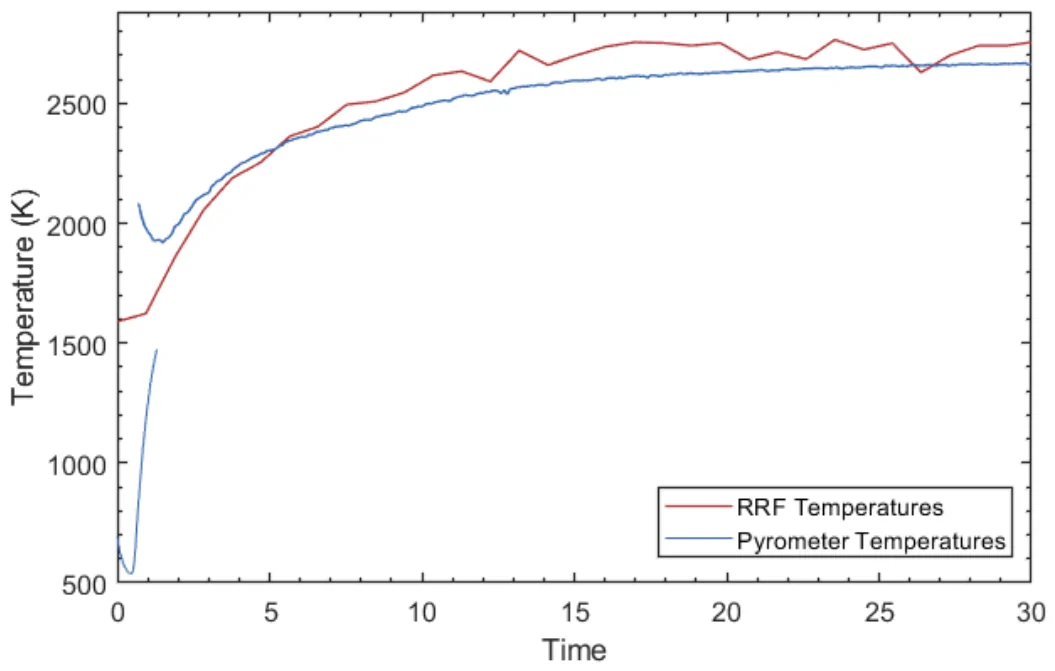
Comparison between temperatures retrieved using the RRF method and the reference pyrometer measurements. The discontinuity in the reference data results from transitioning between the operating ranges of the two pyrometers.

The improved temperature retrieval is shown in Figure 14, where the temperatures retrieved using the RRF method are compared with the pyrometer measurements collected during the experiment. In contrast to the LSEC results, the RRF method remained in close agreement with the reference temperatures throughout the high-temperature portion of the experiment.

The quantitative agreement between the temperatures and spectral emissivities retrieved using the RRF method and the corresponding reference measurements is summarized in Table 1. The reported error metrics include the mean absolute error (MAE), root mean squared error (RMSE), and mean absolute percentage error (MAPE), defined as

(14) $$\mathrm{MAE}=\frac{1}{N}\sum_{j=1}^{N}\left|y_{j}-{\hat{y}}_{j}\right|,$$

(15) $$\mathrm{RMSE}=\sqrt{\frac{1}{N}\sum_{j=1}^{N}{\left(y_{j}-{\hat{y}}_{j}\right)}^{2}},$$

(16) $$\mathrm{MAPE}=\frac{100\%}{N}\sum_{j=1}^{N}\left|\frac{y_{j}-{\hat{y}}_{j}}{y_{j}}\right|,$$

where $y_{j}$ and ${\hat{y}}_{j}$ are the reference and retrieved values for the *j*-th data point, respectively, and *N* is the total number of data points.

The RRF method retrieved temperatures with a mean absolute percentage error of 3.05% and spectral emissivities with a mean absolute percentage error of 3.13%. Although the temperature error may be significant for applications requiring precise absolute temperature measurements, the materials examined in this study exhibited stabilization temperatures differing by several hundred kelvin. More importantly, spectral emissivity remains the primary property used for material characterization because it is an intrinsic material property. The close agreement between the retrieved spectral emissivity and the SOC-100 reference measurements demonstrates that the RRF method accurately recovered the spectral behavior of the carbon phenolic sample under the experimental conditions considered in this work.

**Figure 15:**
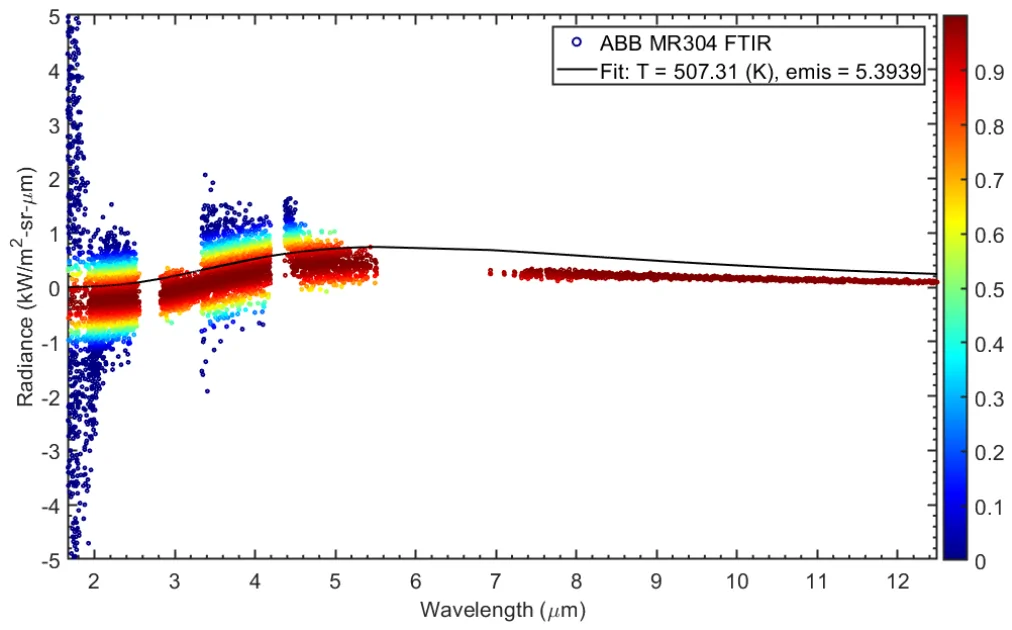
This figure shows the RRF algorithm’s failure when fitting a gray body Planckian curve to data that does not appear to have a blackbody peak, resulting in the algorithm outputting a best-fit gray body emissivity of 5.3939. For the purpose of illustrating the algorithm’s failure at low temperatures, the physical emissivity constraint (0 < ϵ < 1) was disabled when generating this figure. As a result, the algorithm converged to a nonphysical emissivity value greater than one, demonstrating that the gray body fit is no longer valid under these conditions. This behavior is due to the scene radiance most likely peaking around 5.51–6.93 μm. However, that portion of the spectrum does not contain reliable FTIR data because of atmospheric absorption, resulting in the lack of data points within that spectral region. It should be noted that the colorbar indicates the weights assigned to each data point.

One limitation of the RRF method was observed at lower temperatures, where the blackbody peak shifts into spectral regions excluded from the analysis because of atmospheric absorption. As the sample is beginning to heat up, while at temperatures below 1000 Kelvin, the spectral radiance of the scene does not take the shape of a Planckian gray body very well. The gray body fit becomes unreliable because the peak of the Planck radiance occurs within an excluded spectral region, preventing the algorithm from accurately constraining the temperature and resulting in nonphysical emissivity values. Wien’s Displacement Law states that radiation of a blackbody peaks at

(17) $$\lambda_{peak}=\frac{2898\;\mu m\cdot K}{T},$$

placing a large portion of the temperature measurements done with the SOC-100, from 400 K to 600 K, into the region in which the FTIR is ineffective. The peak radiance for a blackbody at these low temperatures lies within a region of atmospheric absorption. To illustrate the failure, the emissivity constraint was removed, and the results are shown in Figure 15. This figure shows the lack of data points where the algorithm is attempting to fit the peak of a Planckian curve. This results in inaccurate temperature and emissivity readings, as shown by the best fit gray body emissivity of around 5.4 in Figure 15.

The results presented in this section demonstrate that the Robust Ratio Linear Fitting method provides accurate temperature and spectral emissivity retrieval for the high-temperature laboratory hyperspectral measurements considered in this study. The method remained effective for temperatures exceeding approximately 1000 K, where the peak of the Planck radiance remained within the measured spectral range. At lower temperatures, the peak shifts into spectral regions excluded because of atmospheric absorption, preventing the gray body fit from being adequately constrained. Although the practical upper temperature limit of the method has not yet been established, the RRF algorithm remained effective throughout the experimental temperature range approaching 2500 K. Future experiments conducted at higher temperatures will be used to determine its operational limits.

## 5. Conclusions

This work presented the Robust Ratio Linear Fitting method, a temperature and emissivity separation algorithm developed for high-temperature laboratory hyperspectral FTIR measurements. Unlike many existing temperature and emissivity separation methods, which were developed for multispectral remote sensing applications requiring extensive atmospheric compensation, the RRF method was designed specifically for laboratory measurements where the optical path length is short but increased measurement uncertainty is present within the short-wavelength portion of the measured spectrum.

The RRF method was evaluated using radiance measurements collected from a carbon phenolic sample heated by a plasma torch to temperatures approaching 2500 K. Retrieved temperatures were compared with reference pyrometer measurements, while retrieved spectral emissivities were compared with a high-accuracy SOC-100 spectral emissivity database. The algorithm retrieved temperatures with a mean absolute percentage error of 3.05% and spectral emissivities with a mean absolute percentage error of 3.13%, demonstrating close agreement with the available reference measurements.

Comparison with the Linear Spectral Emissivity Constraint method showed that increased measurement uncertainty within the short-wavelength portion of the MR304 FTIR spectrum can significantly affect temperature retrieval for high-temperature measurements. By incorporating robust regression into the gray body fitting procedure, the RRF method reduces the influence of wavelength samples exhibiting increased measurement uncertainty while still utilizing the information contained within those measurements during the temperature optimization. Under the experimental conditions considered in this work, this approach produced more consistent temperature retrievals and corresponding spectral emissivity estimates than the LSEC method.

The RRF method also exhibits several limitations. At temperatures below approximately 1000 K, the peak of the Planck radiance shifts into spectral regions excluded from the analysis because of atmospheric absorption, preventing the gray body fit from being adequately constrained. Consequently, the current implementation is best suited for high-temperature laboratory measurements where the spectral region containing the peak of the thermal emission remains within the usable portion of the measured spectrum.

## 6. Future Work

Although the Robust Ratio Linear Fitting method demonstrated accurate temperature and spectral emissivity retrieval for the experimental conditions considered in this work, several opportunities exist to further evaluate and extend the algorithm.

Future work should first focus on applying the RRF method to a broader range of materials. The current study examined a carbon phenolic sample, but additional experiments involving metals, ceramics, oxides, and other carbon-based materials would provide a more comprehensive assessment of the algorithm’s performance across materials exhibiting different spectral emissivity characteristics.

Additional high-temperature reference measurements would also strengthen future evaluations of the algorithm. Because direct high-temperature spectral emissivity measurements are currently unavailable, the retrieved emissivities were compared with the closest available SOC-100 reference measurements collected at lower temperatures. The development of reliable high-temperature spectral emissivity reference datasets would allow the temperature dependence of emissivity to be investigated directly and provide a more rigorous validation of temperature and emissivity separation algorithms.

The current work also demonstrated that increased measurement uncertainty within the short-wavelength portion of the MR304 FTIR spectrum presents a significant challenge for high-temperature temperature and emissivity separation. Future experiments using alternative viewing geometries, different FTIR systems, or improved instrument configurations with greater short-wavelength sensitivity would help determine the extent to which the current limitations arise from the measurement system rather than the retrieval algorithm itself.

Finally, machine learning techniques represent a promising direction for future research. Machine learning models could be incorporated into the current temperature and emissivity separation framework to improve temperature estimation while preserving the underlying radiative transfer relationships. Rather than replacing the current algorithm, hybrid approaches that combine robust regression with machine learning may further improve retrieval performance for challenging high-temperature laboratory measurements.

## Figures and Tables

**Table 1:** Error Measurements for the Carbon Phenolic Sample.

	MAE	RMSE	MAPE
Temperature	78.67 K	85.99 K	3.05%
Spectral Emissivity	0.0204	0.0230	3.13%

## Data Availability

Data underlying the results presented in this paper are not publicly available at this time but may be obtained from the authors upon reasonable request.

## Abbreviations

The following abbreviations are used in this manuscript:

AFIT	Air Force Institute of Technology
FTIR	Fourier Transform Infrared Spectroscopy
GBE	Gray Body Emissivity
HDR	Hemispherical Directional Reflectance
LSEC	Linear Spectral Emissivity Constraint
MAE	Mean Absolute Error
MAPE	Mean Absolute Percentage Error
RMSE	Root Mean Squared Error
RRF	Robust Ratio Linear Fitting
TIMS	Thermal Infrared Multispectral Scanner
UIUC	University of Illinois Urbana-Champaign

## Appendix A. Uncertainty Sources and Wavelength-Dependent Sensitivity

Several sources of uncertainty influence the temperatures and spectral emissivities retrieved in this study. These sources include the radiometric calibration of the MR304 FTIR, extrapolation of the calibration above the highest blackbody calibration temperature, reduced optical transmission below approximately 2.5 μm, spectrometer noise, transmission through the chamber observation window, residual atmospheric absorption, pyrometer measurement uncertainty, temporal synchronization between the FTIR and pyrometer datasets, and differences in the instrument viewing geometries.

Additional uncertainty is introduced when comparing the FTIR-derived spectral emissivity with the SOC-100 reference measurements. The FTIR viewed the sample at approximately 82° relative to the surface normal, while the maximum SOC-100 measurement angle was 80°. The SOC-100 spectral emissivity at 82° was therefore estimated by linearly extrapolating the measured angular trend between 70° and 80°. The SOC-100 measurements were also collected at substantially lower temperatures than those reached during the plasma heating experiment. Consequently, temperature-dependent changes in the sample, including oxidation or structural changes, may contribute to differences between the reference and retrieved spectral emissivities.

Several of these uncertainty sources are wavelength dependent, systematic, or not independently quantified. A complete numerical propagation of every uncertainty source through the multiwavelength robust regression was therefore not performed. Instead, the following first-order analysis examines how uncertainties in spectral radiance and temperature affect the inferred temperature and spectral emissivity as functions of wavelength.

The wavelength-dependent treatment is useful because both the uncertainty in the measured radiance and the sensitivity of Planck radiance to temperature vary across the measured spectrum. The temperature uncertainty calculated at each wavelength represents the local sensitivity of a temperature estimate to radiance uncertainty at that wavelength. Because the RRF method estimates a single temperature using a robust regression across many wavelength samples, this analysis does not represent the total uncertainty of the final RRF temperature. Instead, it identifies wavelength regions that have the greatest potential to influence the temperature retrieval.

The relationship between spectral radiance, spectral emissivity, and Planck radiance is

(A1) L(λ)=ϵ(λ)B(λ,T),

where

(A2) $$B\left(\lambda ,T\right)=\frac{2hc^{2}}{\lambda^{5}}\frac{1}{\exp\left(\frac{hc}{\lambda kT}\right)-1}.$$

In Equation (A2), *h* is Planck’s constant, *c* is the speed of light, *k* is the Boltzmann constant, λ is wavelength, and *T* is temperature.

### Appendix A.1. Wavelength-Dependent Temperature Sensitivity

For a fixed wavelength and constant emissivity, the first-order uncertainty in temperature resulting from an uncertainty in spectral radiance is

(A3) $$\Delta T\left(\lambda \right)=\left|\frac{\partial T}{\partial L}\right|\Delta L\left(\lambda \right),$$

where ΔL(λ) represents the wavelength-dependent spectral radiance uncertainty.

Differentiating Equation (A1) with respect to temperature gives

(A4) $$\frac{\partial L}{\partial T}=\epsilon \left(\lambda \right)\frac{\partial B(\lambda ,T)}{\partial T}.$$

The inverse sensitivity is therefore

(A5) $$\frac{\partial T}{\partial L}=\frac{1}{\epsilon \left(\lambda \right)\frac{\partial B(\lambda ,T)}{\partial T}}.$$

Let

(A6) $$x=\frac{hc}{\lambda kT}.$$

Planck radiance can then be written as

(A7) $$B\left(\lambda ,T\right)=\frac{2hc^{2}}{\lambda^{5}}\frac{1}{e^{x}-1}.$$

Differentiating Planck radiance with respect to temperature gives

(A8) $$\frac{\partial B(\lambda ,T)}{\partial T}=\frac{2hc^{2}}{\lambda^{5}}\frac{xe^{x}}{T{\left(e^{x}-1\right)}^{2}}.$$

Substituting Equation (A8) into Equation (A3) gives

(A9) $$\Delta T\left(\lambda \right)=\left|\frac{1}{\epsilon \left(\lambda \right)\frac{2hc^{2}}{\lambda^{5}}\frac{xe^{x}}{T{\left(e^{x}-1\right)}^{2}}}\right|\Delta L\left(\lambda \right).$$

Equation (A9) describes the local sensitivity of temperature to radiance uncertainty at each wavelength. The value of ΔT(λ) depends on both the wavelength-dependent measurement uncertainty and the rate at which Planck radiance changes with temperature. Wavelengths with greater radiance uncertainty or lower temperature sensitivity produce larger values of ΔT(λ).

### Appendix A.2. Wavelength-Dependent Spectral Emissivity Uncertainty

After the temperature has been estimated, the spectral emissivity is calculated as

(A10) $$\epsilon \left(\lambda \right)=\frac{L(\lambda )}{B(\lambda ,T)}.$$

The first-order uncertainty in spectral emissivity may be expressed in its general form as

(A11) $$\begin{array}{cc} {\left[\Delta \epsilon (\lambda )\right]}^{2}= & {\left[\frac{\partial \epsilon }{\partial L}\Delta L\left(\lambda \right)\right]}^{2}+{\left[\frac{\partial \epsilon }{\partial T}\Delta T\right]}^{2}\\ \; & +2\frac{\partial \epsilon }{\partial L}\frac{\partial \epsilon }{\partial T}\mathrm{Cov}\left(L,T\right), \end{array}$$

where Cov(L,T) represents the covariance between the radiance and temperature uncertainties.

The required partial derivatives are

(A12) $$\frac{\partial \epsilon }{\partial L}=\frac{1}{B(\lambda ,T)}$$

and

(A13) $$\frac{\partial \epsilon }{\partial T}=-\frac{L(\lambda )}{B^{2}\left(\lambda ,T\right)}\frac{\partial B(\lambda ,T)}{\partial T}.$$

Substitution of Equation (A8) gives

(A14) $$\frac{\partial \epsilon }{\partial T}=-\frac{L(\lambda )}{B^{2}\left(\lambda ,T\right)}\frac{2hc^{2}}{\lambda^{5}}\frac{xe^{x}}{T{\left(e^{x}-1\right)}^{2}}.$$

When the spectral radiance uncertainty and temperature uncertainty are determined independently, the covariance term in Equation (A11) may be neglected. Under this assumption, the wavelength-dependent emissivity uncertainty is

(A15) $$\Delta \epsilon \left(\lambda \right)=\sqrt{{\left[\frac{\Delta L(\lambda )}{B(\lambda ,T)}\right]}^{2}+{\left[\frac{L(\lambda )}{B^{2}\left(\lambda ,T\right)}\frac{2hc^{2}}{\lambda^{5}}\frac{xe^{x}}{T{\left(e^{x}-1\right)}^{2}}\Delta T\right]}^{2}}.$$

Equation (A15) describes the combined first-order effects of independent radiance and temperature uncertainties on the retrieved spectral emissivity. If ΔT is calculated directly from the same radiance uncertainty used for ΔL(λ), the two quantities are correlated and the covariance term should not be neglected. In that case, Equation (A11) provides the more appropriate general expression.

This analysis characterizes the wavelength dependence of the retrieval sensitivity but does not include the complete behavior of the RRF algorithm. The RRF temperature is determined using a robust regression across thousands of spectral samples, with atmospheric absorption bands excluded and lower weights assigned to wavelength samples with larger residuals. Therefore, the final RRF uncertainty depends on the combined spectral information, robust weights, spectral mask, and correlations among wavelength samples. A complete uncertainty propagation would require perturbing the full measured radiance spectrum and repeating the complete RRF retrieval for each perturbed realization.
